# Treatment of a Refractory Skin Ulcer Using Punch Graft and Autologous Platelet-Rich Plasma

**DOI:** 10.1155/2016/7685939

**Published:** 2016-02-17

**Authors:** Mauro Carducci, Marcella Bozzetti, Marco Spezia, Giorgio Ripamonti, Giuseppe Saglietti

**Affiliations:** ^1^Department of Dermatologic Surgery, Centro Ortopedico di Quadrante Hospital, 28882 Omegna, Italy; ^2^Department of Orthopedics, Centro Ortopedico di Quadrante Hospital, 28882 Omegna, Italy; ^3^Department of Medicine, Centro Ortopedico di Quadrante Hospital, 28882 Omegna, Italy; ^4^Department of Metabolic Disease and Diabetology, ASL VCO, 28925 Verbania, Italy

## Abstract

*Background*. Chronic ulceration of the lower legs is a relatively common condition amongst adults: one that causes pain and social distress and results in considerable healthcare and personal costs. The technique of punch grafting offers an alternative approach to the treatment of ulcers of the lower limbs.* Objective*. Combining platelet-rich plasma and skin graft enhances the efficacy of treating chronic diabetic wounds by enhancing healing rate and decreasing recurrence rate. Platelet-rich plasma could, by stimulating dermal regeneration, increase the take rate after skin grafting or speed up reepithelialization.* Methods and Materials*. The ulcer was prepared by removing fibrin with a curette and the edges of the ulcer were freshened. The platelet-rich plasma has been infiltrated on the bottom and edges of the ulcer. The punch grafts were placed in 5 mm holes arranged. The ulcer was medicated with hydrogel and a pressure dressing was removed after 8 days.* Results*. After a few days the patient did not report more pain. Granulation tissue appeared quickly between implants. Most of the grafts were viable in 2-3 weeks. The grafts gradually came together to close the ulcer and were completed in four months.

## 1. Introduction

Chronic ulceration of the lower legs is a relatively common condition amongst adults: one that causes pain and social distress and results in considerable healthcare and personal costs [1, 2].

The 3 main types of lower extremity ulcers are venous, arterial, and neuropathic. Venous ulcers constitute the majority of all leg ulcers, whereas foot ulcers are more likely to be due to arterial insufficiency or neuropathy. Up to 80% of leg ulcers are caused by venous disease.

Treatment goals for patients with chronic venous insufficiency include reduction of edema, alleviation of pain, improvement of lipodermatosclerosis, healing of ulcers, and prevention of recurrence. Better understanding of the pathophysiology of venous disease and leg ulceration has in turn suggested new approaches to the management of ulcer disease with new types of wound dressings, compression bandages, topical and systemic therapeutic agents, and surgical modalities [3].

Surgical treatment of venous ulcers may be directed toward modifying the cause of venous hypertension or treating the ulcer itself by a graft. There are no specific indications for when skin grafting for lower extremity ulcers should be used. Larger or refractory ulcers are two instances when grafting should be considered. Even if grafts do not take, they likely stimulate wound that may rapidly and markedly relieve pain [4].

Wound healing is a complex process mediated by interacting molecular signals involving mediators and cellular events. Platelets play two important roles in wound healing: hemostasis and initiation of wound healing. After platelet activation and clot formation, growth factors are released from granules located in the thrombocyte cell membrane.

Growth factors work as biologic mediators to promote cellular activity by binding to specific cell surface receptors [5, 6].

Recent studies have found that autologous platelet concentrate with growth factors (APGF) or platelet-rich plasma (PRP) may accelerate wound healing [7].

There is also indication that platelet-rich plasma has infection-fighting properties and it has been proposed as an adjunct for the treatment of diabetic foot ulcers [8, 9]. Platelet-rich plasma is most often mixed with thrombin before application in order to generate a fibrin gel, often called platelet gel, and platelet-growth-factors-rich exudates [10].

The technique of punch grafting offers an alternative approach to the treatment of ulcers of the lower limbs. The method was first described by Reverdin [11] and later developed by Davis [12]; it is a relatively simple technique without immobilization of the patient, suited to the treatment of outpatients [13].

Good results were obtained regarding the reduction of pain and improvement in quality of life [14]. Similar results were obtained more recently by Swedish authors [15].

The method is also taken into consideration in the past American guidelines for the management of ulcers [16].

Combining platelet-rich plasma and skin graft enhances the efficacy of treating chronic diabetic wounds by enhancing healing rate and decreasing recurrence rate [17].

Autologous platelet gel could help to create a vascularised matrix, aiding the success of skin grafting in patients. Platelet-rich plasma could, by stimulating dermal regeneration, increase the take rate after skin grafting or speed up reepithelialization [18].

## 2. Case Presentation

**Figure 1:**
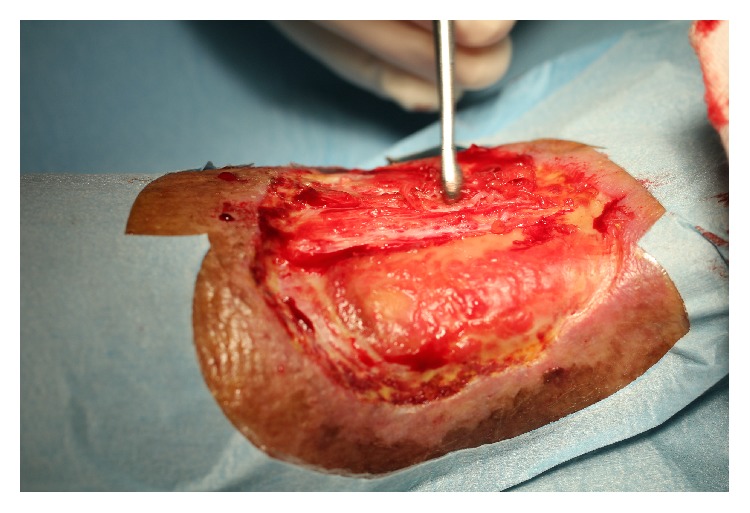
Right ankle: the ulcer was prepared by removing fibrin with a curette and the edges of the ulcer were freshened.

**Figure 2:**
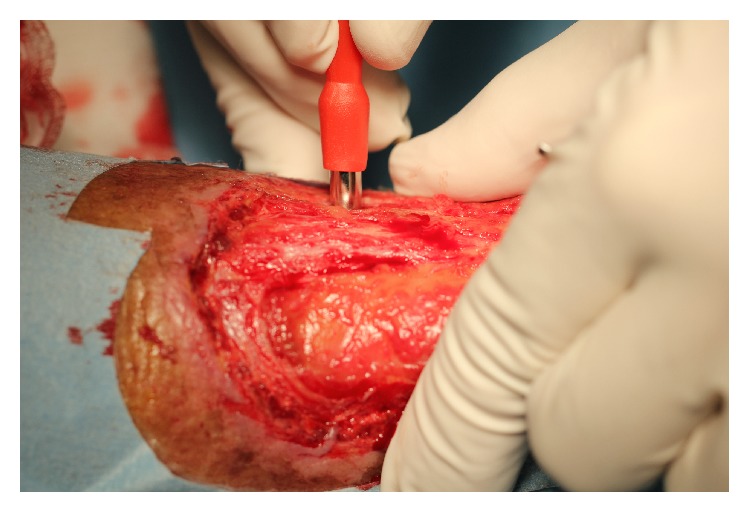
Holes were drilled in the ulcer using a punch 5 mm while maintaining a distance of one centimeter between the holes.

Patient is a male, 77 years old, diabetic, and treated with oral hypoglycemic drugs and antihypertensive therapy, with good overall clinical condition. A mixed arterial and venous wide ulcer was localized in left ankle from three years. The patient has been followed up by an outpatient service of diabetes with topical treatments and advanced dressing with mixed results. The ulcer was extended over 50% of the circumference of the left ankle. The Achilles tendon was partially exposed. The patient was subjected to periodic Doppler ultrasound to check the venous and arterial circulation. The foot pulses were retained. The lesion was painful, in particular in the night hours. The ulcer was prepared by removing fibrin with a curette and the edges of the ulcer were freshened (Figure 1). Local anesthesia has been practiced around the ulcer and in the donor site graft. Platelet rich plasma was prepared using gravitational platelet heparinazed system. Briefly, the patient's phlebotomization consisted of 27 mL of whole blood and was drawn from the median cubital vein with a 21-gauge needle. One 30 mL syringe with 3 mL of anticoagulant citrate dextrose (ACD) solution formula 3 cc of platelet-rich plasma was extracted. Holes were drilled in the ulcer using a punch 5 mm while maintaining a distance of one centimeter between the holes (Figure 2).

**Figure 3:**
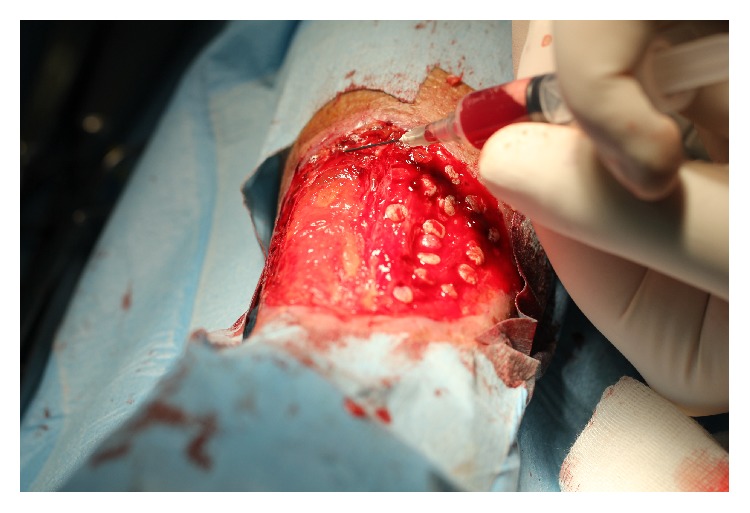
The platelet-rich plasma has been infiltrated on the bottom of the punch graft and edges of the ulcer.

The platelet-rich plasma has been infiltrated on the bottom and edges of the ulcer (Figure 3).

From the donor sites, skin was harvested with a 6 mm biopsy punch. The grafts were removed with scissors and tweezers and placed on sterile, saline-moistened cotton dressings; the punch grafts were taken from the arm. The grafts were placed in 5 mm holes arranged. Local anesthesia has been practiced in the ulcer compared to the donor site graft. The ulcer was medicated with hydrogel and a pressure dressing was removed after 8 days, in successive weeks the wound was medicated with paraffin gauze.

If the ulcers are very extensive, you can perform surgery in 2 or 3 steps (Figure 5).

**Figure 4:**
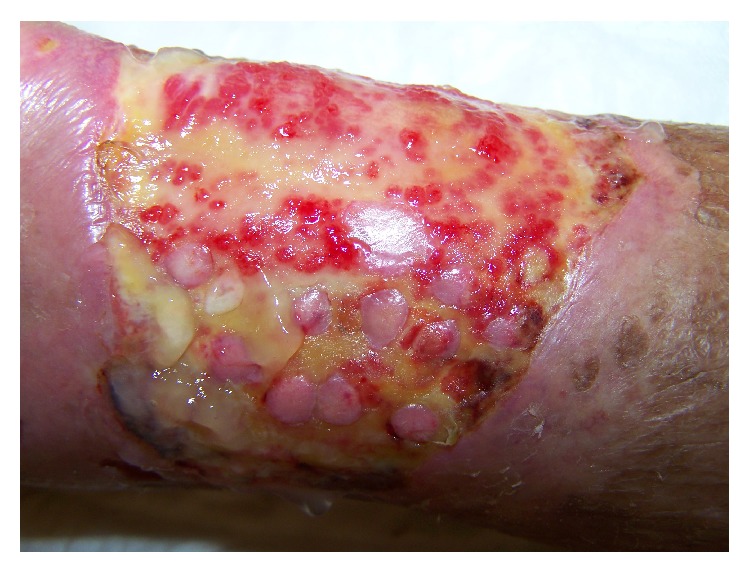
Epithelialization of the edge and epithelial islands inside the ulcer, one month later surgery treatment with punch grafting and platelet-rich plasma.

**Figure 5:**
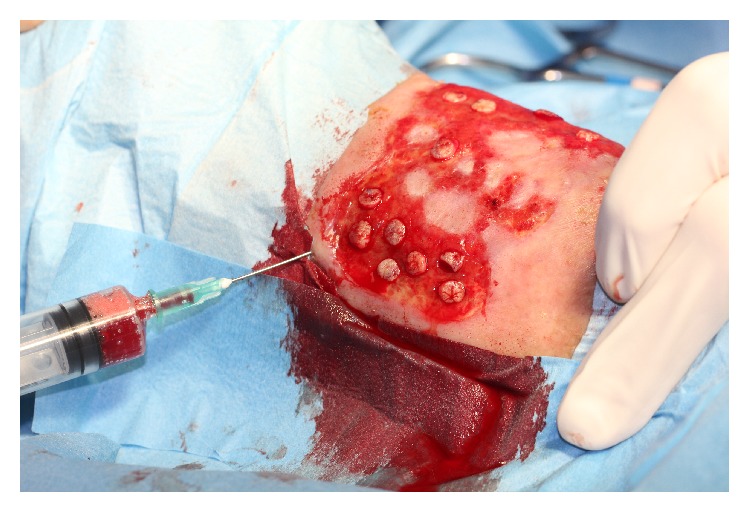
After 2 months we repeat intervention in areas not treated the first time. You can see the islands of reepithelialization of the previous treatment.

**Figure 6:**
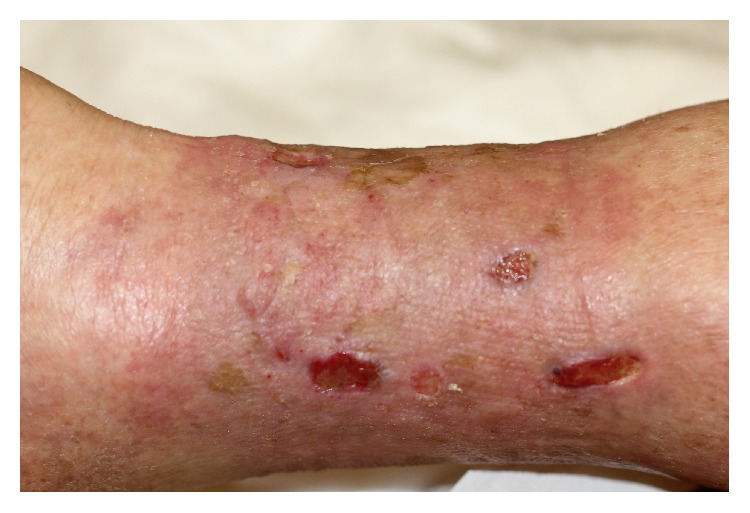
Complete reepithelialization after four months confirmed in follow-up, one year later.

After a few days the patient no longer reported pain. Granulation tissue appeared quickly between the grafts. Most of the grafts took root after 2-3 weeks and started the development of the islands of epithelial tissue that gradually came together to close the ulcer (Figure 4). The process of reepithelialization was completed in four months (Figure 6). The conditions of life of the patient are significantly improved by the absence of pain and the limited number of dressings.

## 3. Conclusion

The repair of leg ulcers causes discomfort to the patient with a high social cost.

The rapid healing of ulcers improves the patient's life by reducing the inconvenience of constant medication. The use of punch graft is a simple technique manageable even on an outpatient that quickly reduces the pain and eases the reepithelialization ulcer. The use of punch graft increases the possibility that epithelial cells are developed and the fragmentation of the islands reduces the risk of failures (Figure 2).

We believe that the combined technique can benefit from the advantages of both methods.

Autologous platelet gel could help to create a vascularised matrix, aiding the success of skin grafting in patients. Platelet-rich plasma could, by stimulating dermal regeneration, increase the take rate after skin grafting or speed up reepithelialization. The repair process is accelerated by the use of platelet-rich plasma. The technique has been applied to a limited number of patients, but they all had an important reduction in pain and all ulcers treated were completely closed in a time ranging between 3 and 6 months. The follow-up at one year confirms the result (Figure 6).

We treated 9 patients with 11 ulcers resistant to conventional topical therapies. Three patients with 5 ulcers are still in follow-up.
